# Trait-Anxious People Take Longer to Search for Happy Faces in the Presence of Neutral and Fearful Distractors

**DOI:** 10.1007/s43076-023-00305-8

**Published:** 2023-06-22

**Authors:** Eda Tipura, David Souto, Elaine Fox

**Affiliations:** 1 Laboratory of Behavioral Neurology and Imaging of Cognition, Department of Neuroscience, University Medical Center, University of Geneva, Geneva, Switzerland; 2 Faculty of Psychology and Educational Sciences, University of Geneva, Geneva, Switzerland; 3 School of Psychology and Vision Sciences, University of Leicester, Leicester, UK; 4 School of Psychology, University of Adelaide, Adelaide, Australia

**Keywords:** Trait anxiety, Context sensitivity, Eye tracker, Positive emotions, Attentional bias

## Abstract

A large body of evidence suggests that processing of affective information is typically disrupted in anxiety. It has also been hypothesized that anxious individuals are less able to evaluate contextual cues and to respond in an adaptive way to stress. In the present study, 25 participants (16 females; 9 males) scoring high (scores of 45 or above) and 26 participants (13 females; 13 males) scoring low (scores of 35 and below) on a standardized measure of trait anxiety performed an emotion search task to investigate attentional biases when the task provides an explicit emotional context. An emotional context was set in each block by asking participants to look as quickly as possible at a face expressing a specific emotion, while eye movements were being recorded. On each trial, two faces appeared, one of them expressing the target emotion and the other one expressing a distractor emotion. High trait-anxious participants showed slower response times (time to look at the instructed emotion), regardless of the affective context, compared to the control group. Additionally, we found slower responses to happy faces (positive context) in the anxious group in the presence of neutral and fearful distractors. Cognitive control may therefore be disrupted in anxiety, as anxious people take longer to process (search for) happy faces, presumably because attentional resources are drawn by neutral and fearful distractors. Those differences were not observed in a simple reaction times task, which suggests that attentional biases, and not differential processing of low-level facial features, are responsible for those differences.

## Introduction

Anxiety-related attentional biases towards threat-related stimuli are often reported (e.g. Fox, 2002; MacLeod & Mathews, 1988), typically using a version of a dot-probe task (MacLeod et al., 1986). Briefly, in this task, two images (e.g. words or faces) appear simultaneously and differ in the emotion they express (e.g. an angry facial expression alongside a neutral expression). These images are then replaced by a neutral probe that the participants must detect. The trial is congruent if the probe replaces the emotional face and incongruent if it appears at the location of the neutral face. The difference in response times between congruent and incongruent trials is taken to reflect attentional bias towards negative emotions in anxious individuals (e.g. Cannito et al., 2020; Clauss et al., 2022; Egloff & Hock, 2001; Eysenck et al., 2007; Fox et al., 2007; Mathews & MacLeod, 1985; Okon-Singer, 2018). Importantly, the mechanisms underlying the effects of state and trait anxiety on attention differ. While state anxiety refers to a transitory state, trait anxiety is a more stable measure of emotional response to different types of situations or events (Spielberger, 1983). Pacheco-Unguetti et al. (2010) showed a double dissociation of the effect of anxiety on attention: while state anxiety was linked to an over-functioning of the alerting and orienting components of attention, trait anxiety was characterized by decreased executive control. In trait anxiety, this decrease in executive control is associated with a reduction in the recruitment of attentional control mechanisms in prefrontal brain regions (Bishop, 2008; Modi et al., 2015). Using EEG methods, it has been reported that high trait anxiety modulates electrophysiological markers of attention in the dot-probe paradigm (Fox et al., 2008). However, some studies have failed to find evidence for anxiety-related biases in attention using the dot-probe task (Kruijt et al., 2019). The measurement of attentional biases by means of response times has been questioned recently given that reaction time-based difference scores have been shown to lack reliability (Kappenman et al., 2014; Parsons et al., 2019).

These findings raise concerns about an overreliance on reaction time measures to assess biased attention. Some possible ways to provide more reliable methods to evaluate anxiety-related attentional biases are to use eye-tracking (e.g. Skinner et al., 2018) or electrophysiological (Fox et al, 2008; Tipura & Fox, 2020) methodology. Not only may such methods provide a more accurate assessment of attentional bias, but eye-tracking and electroencephalography also provide a window into the dynamic processes that underpin visual attention across an entire task (e.g. Sharpe et al., 2017). To illustrate, a recent meta-analysis of eye-tracking studies showed attentional biases for pain-related words and pictures in terms of both probability of first fixation and overall dwell time on pain-related images (Jones et al., 2020). Anxiety-related attentional biases towards threat using eye-tracking methodology have also been reported in generalized anxiety disorder (e.g. Mogg et al., 2000) and show an increase in viewing time on threatening images in state (Quigley et al., 2012) and trait anxiety (Veerapa et al., 2020) as well as a difficulty in disengaging from threatening body postures in trait anxiety (Azarian et al., 2016). Biases in the attentional processing of threat-related information can be understood in the context of attentional control theory (ACT; Derakshan & Eysenck, 2009), which proposes that anxiety tips the balance to stimulus-driven processes so that highly salient affective cues will override top-down control mechanisms, which would explain enhanced attentional orienting towards emotional faces in anxious individuals.

Attentional processes are also of interest in anxiety and related states as they are likely to underlie the emotion regulation impairments often reported in high-anxious individuals (Campbell-Sills & Barlow, 2007; Mennin et al., 2007). For example, a study that attempted to reduce the degree of attentional biases using a cognitive training technique led to changes in reappraisal processes which in turn led to reduced negative emotions (Sanchez et al., 2016). The potential of cognitive training techniques, such as ‘attention bias modification’ (MacLeod et al., 2002; Van Bockstaele et al., 2019), to modify emotion regulation processes has obvious therapeutic and preventative potential. However, it is important to note that contextual factors can have profound impact on the efficacy of any particular emotion regulation strategy (e.g. Aldao, 2013; Bonanno & Burton, 2013; Troy et al., 2013). Rather than assuming that specific strategies are always adaptive or maladaptive, it is now widely accepted that the *flexible* use of different strategies and their relevance to a specific context is optimal (Aldao, 2013; Bonanno & Burton, 2013). It is therefore vital to take contextual factors into account when evaluating anxiety-related biases in attention and deciding whether the modification of such biases might be of benefit.

We therefore think that it is useful to consider the nature of anxiety-related attentional biases within a framework of regulatory flexibility (e.g. Bonanno & Burton, 2013; Parsons et al., 2016). Bonanno and Burton (2013) have provided a useful framework and proposed that ‘regulatory flexibility’ is characterized by three sequential components: context sensitivity, repertoire and feedback. When a stressful event occurs and people must choose between different regulation strategies, they will first evaluate demands and opportunities in the environment (context sensitivity). This first component relies on an ability to read and decode contextual cues. Once contextual cues are decoded, the next step is to select the regulatory strategy among all the strategies available in a person’s repertoire. Finally, through a feedback process, the regulation strategy is monitored and modified if needed, resulting in either adjustment, maintenance or cessation. At the end of the process, the re-evaluation of demands and opportunities might be necessary so that optimal regulation will be the one engaging the most flexible pattern. It has been suggested that psychopathological states such as depression or anxiety may reflect an insensitivity in the processing of context (Bylsma et al., 2008; Larson et al., 2007; Lenzo et al., 2021). It is clear that if attentional processes interfere with the processing of context right at the outset, the entire regulatory process will be disrupted.

If the emotional context is incorrectly decoded, then this will affect the other components of regulatory flexibility, and therefore, emotion regulation generally will be affected. Given the close association between the use of emotion regulation strategies and psychopathology (Dixon-Gordon et al., 2015), we propose that it may be informative to investigate negative attentional biases within a framework of regulatory flexibility. Indeed, one of the challenges of investigating emotion perception in laboratory settings is the absence of contextual features that allow participants to evaluate the extent to which an emotional stimulus (like a face) is relevant to the task or not. While different types of stimuli (scenes, bodies, faces or words) have been used to indicate specific emotions, these stimuli are quite often taken out of their usual context, making it difficult to translate the findings to more realistic situations (Barrett et al., 2011). In line with this idea, the constructed emotion approach (Barrett et al., 2011) assumes that context is vital to build an emotion. Without context, it is difficult to assess emotions, especially in laboratory settings. For instance, structural features of a face might not be sufficient for decoding emotions. Therefore, it is important to understand the effect of emotional context on attentional biases in the anxious population.

The current study was inspired by the effects of threat-related stimuli on attentional processes in trait-anxious individuals. We sought to test whether these processes are affected by the decoding of emotional contextual cues by using eye movement measures. We operationalized the notion of emotional context by asking participants to search for specific emotions over several trials. Specifically, participants were instructed to search for, and fixate as fast as possible, a face displaying a given emotion in the presence of a distractor emotion. An instruction that monetary compensation will depend on their performance added pressure to perform and acted as a stressor in our study. We used gaze as an overt measure of selective attention, with differences in response times (time to fixate the target emotion) indicative of differences in the processing of different emotional faces. The idea is that if anxious people are less sensitive to context—as determined by the ‘target’ facial expression—they would be more prone to distraction from irrelevant facial expressions. In contrast, if lower anxiety is associated with an enhanced ability to decode context, then there should be less interference from an irrelevant distractor.

## Method

### Participants

Fifty-five participants from the University of Oxford and the local community were preselected based on their scores on the trait scale of the State-Trait Anxiety Inventory (STAI; Spielberger, 1983) which was completed online before the experiment. There was a maximum of 1-week interval between the assessment of anxiety and the participation in the search task. We selected twenty-eight participants scoring high (scores of 45 or above) and twenty-eight participants scoring low (scores of 35 and below) on the STAI (Fox et al., 2007; Georgiou et al., 2005). Following the eye movements artefact rejection procedure and the suppression of incorrect trials, 1 participant was excluded due to a high percentage of incorrect trials (> 50%) and 3 participants were excluded from the analyses due to a high percentage of trial loss during the artefact rejection procedure (> 50%). The final sample consisted of 51 participants. The trait-anxious group consisted of 16 females and 9 males with a mean age of 25.44 years (*SD* = 5.07) and had a mean of 49.28 (*SD* = 4.91) on the STAI-T. The control group consisted of 13 females and 13 males with a mean age of 24.62 years (*SD* = 4.86) and a mean of 28.11 (*SD* = 4.9) on the STAI-T. In the literature and in our sample the STAI-T shows excellent consistency (Cronbach’s alpha is respectively .94 and .95). Participants had a normal or a corrected-to-normal vision. They were paid £10 per hour for their participation.

Prior to the experiment, participants completed the Context Sensitivity Index (CSI; Bonanno et al., 2020), which is a 20-item questionnaire reflecting cue presence (sensitivity to the presence of contextual cues) and cue absence (sensitivity to the relative absence of cues). The index of cue absence has been inversely associated with anxiety, namely more anxious people are less able to detect the absence of contextual cues (Bonanno et al., 2020). This measure has been proposed to best account for context sensitivity, as experimental measures tend to confound perception and response to context (CSI; Bonanno et al., 2020). We used this measure to test whether anxious participants are less inclined to detect contextual cues and determine cue absence, which would replicate the original findings (Bonanno et al., 2020). In our sample, the correlation between trait anxiety and cue presence was small (*r*(49) =  − 0.0002, *p* = 0.99) and the correlation between trait anxiety and cue absence was significant (*r*(49) =  − 0.52, *p* < 0.001), confirming the observations made by Bonanno et al. (2020).

An a priori power analysis was performed using G*Power 3.1.9.4 (Faul et al., 2007) with two groups and three measurements for a medium effect size of 0.25, using ‘*F*-tests’ and ‘ANOVA: repeated measures, within-between interaction’, and an alpha of 0.05. A total sample of 44 participants was required to achieve a power of 0.95.

### Stimuli and Experimental Procedure

#### Stimuli

Stimuli were photographs of 10-male and 10-female grayscale fearful, happy and neutral facial expressions taken from the Radboud faces database (Langner et al., 2010). The borders of the faces were removed to avoid the influence of features such as the hair or ears. All the stimuli were created using Adobe Photoshop which allowed us to equate gray levels across all categories. Additionally, the SHINE toolbox was used to control for low-level properties of the stimuli across all categories and adjust mean gray level and contrast between the different faces (Willenbockel et al., 2010).

#### Experimental Procedure

**Fig. 1:**
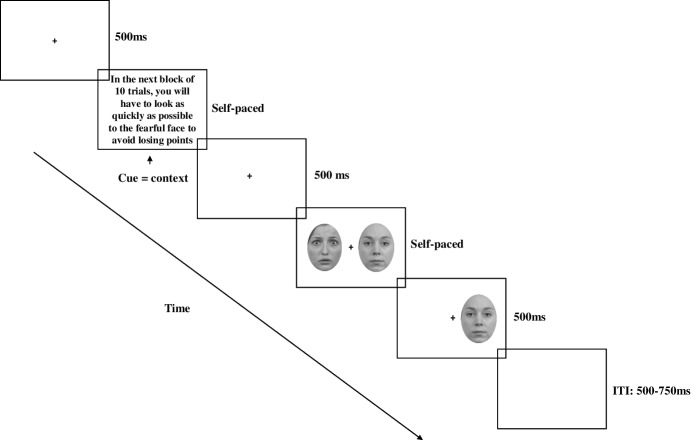
Experimental procedure

The experiment was divided in 72 blocks of 10 trials, each block representing a specific context. Each block started with the following instruction: ‘In the next block of 10 trials, you will have to look as quickly as possible to the fearful / happy / neutral face to avoid losing points’. This instruction defined the emotional context: when instructed to look at the fearful face the context was ‘fear’, when instructed to look at the happy face the context was ‘happy’, when instructed to look at the neutral face the context was ‘neutral’. Next, two faces were presented side by side. One of the faces was congruent with the emotional context (either fearful, happy or neutral) and the other face expressed one of the two other emotions and acted as a distractor. The task of the participant was to look as quickly as possible towards the face expressing the emotion indicated by the previous instruction (the emotional context). Once the gaze of the participant was on a face, the face disappeared. This acted as a feedback for the participant. If the face expressing the emotion that matched the context disappeared, then it was to be considered a correct response. If the other face disappeared, it was to be considered an incorrect response (see Fig. 1).

The instruction acted as a mild stressor since participants were told that the number of correct responses would define the amount of money they receive. But in fact, all the participants were paid £10 per hour for their participation.

#### Simple Reaction Time Control with Trait-Anxious and Control Groups Study

Given that the three emotions differ in low-level features due to appearance of some facial features such as the teeth, to make sure that the results were not due to these low-level properties and only reflect orientation to a direction due to these low-level properties, we conducted an additional experiment with one face appearing on the right or left side of the screen and asked the participants to stare at the face as quickly as possible. The face was expressing either happy, neutral or fearful emotion and was composed of 90 trials randomly assigned to one of the emotions. Reaction times were analysed using a repeated measures ANOVA with emotion (fear/happy/neutral) as the within-subject factor and group (trait-anxious/control) as the between-subject factor. We also conducted a Bayesian repeated measures ANOVA to ensure that there were no differences between emotions. For the Bayesian analyses, models were compared to the null model with the default settings implemented in JASP (JASP Team, 2023).

The study was approved by the Central University Research Ethics Committee at the University of Oxford (CUREC Approval Reference: R63832/RE002) and all participants signed an informed consent form before participating. The study was performed in agreement with the Declaration of Helsinki.

### Eye-Tracking Apparatus

We used a Tobii Pro TX300 (Stockholm, Sweden) eye-tracking system (screen + eyetracker). Gaze measurements are based on the pupil centroid (dark-pupil tracking) with a sampling frequency of 300 Hz. The TFT screen resolution was set to 1980 × 1024 pixels at 60 Hz. A custom MATLAB script was used to display the stimuli with the Psychtoolbox-3 (Kleiner et al., 2007) and interface with the eyetracker with the Tobii Pro Software Development Kit (SDK, downloaded 12/06/2019). The participants’ head rested on a chinrest at 60 cm from the screen centre.

### Analyses

#### Eye Movements Pre-processing

Saccade and fixation identification used the default settings of the Tobii Pro I-VT filter (Komogortsev et al., 2010). We excluded saccades which direction was opposite to the targets’; we also excluded saccades which duration was below 10 ms and those which amplitude was below a 1.5 degrees of visual angle. Finally, there were some rare trials where a saccade could not be detected within the 5 s of the presentation of the face. This amounted to 2.4% of trials. Response times (time to look at the face displaying the instructed emotion) shorter than 200 ms and longer than 1000 ms were excluded from the analysis.

#### Statistical Analysis

We did separate analyses for the median time to focus (the time between the appearance of the two faces and the saccade made by the participant) and accuracy (if the participant looked at the emotional expression as instructed in the block the response was correct; incorrect if the participant looked at the distractor) in the three contexts (i.e. instructions to orient towards specific emotional expressions) and for transitions between the contexts. We also did separate analyses for the emotional context and for the distractor (the distractor is the other face displayed during the trial, which always expresses a distinct emotion than the one instructed). We analysed the percentage of incorrect responses and time to focus as a function of emotional context (fear/happy/neutral) and distractor (when the distractor was fear, happy or neutral) in trait-anxious and control (low anxiety) participants.

A repeated measures ANOVA was first performed on the percentage of incorrect responses with emotional context (fear/happy/neutral) as the within-subjects factor and anxiety group (trait-anxious/control) as the between-subjects factor. For the distractor, a repeated measures ANOVA was performed on the percentage of incorrect responses with the distractor valence (fear/happy/neutral) as the within-subjects factor and anxiety group (trait-anxious/control) as the between-subjects factor. The same analyses were performed with time to focus as the dependent variable.

To analyse how transitions between contexts affect response time, we took (individually) the median time to focus within a block of trials for each transition, as defined by the preceding and current emotional context, making six different transitions. A repeated measures ANOVA was performed on time to focus with transitions (happy following fear, happy following neutral, fear following happy, fear following neutral, neutral following fear, neutral following happy) as the within-subjects factor and anxiety group (trait-anxious/control) as the between-subjects factor.

When necessary, adjusted *p*-values were used to control for sphericity. In the ‘Results’ section, partial eta squared (*η*^2^_p_) and Cohen’s *d* are reported as effect sizes with *η*^2^_p_ = 0.01 indicating a small effect, *η*^2^_p_ = 0.06 indicating a medium effect, *η*^2^_p_ = 0.14 indicating a large effect, *d* = 0.2 indicating a small effect, *d* = 0.5 indicating a medium effect and *d* ≥ 0.8 indicating a large effect (Cohen, 1988).

#### Vincent Averaging

**Fig. 2:**
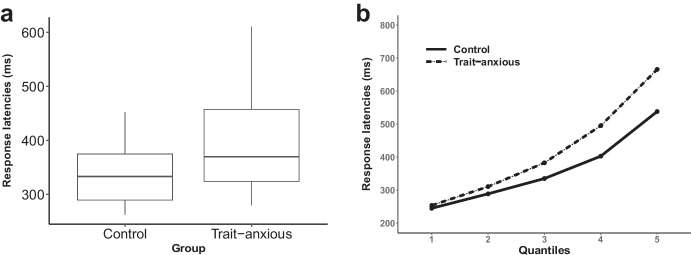
**a **Distribution of response latencies (times to focus; RTs) in each group, showing more variability in the trait-anxious group. **b** Response latencies as a function of quantile and group (dashed line, trait-anxious group; solid line, control group)

To better capture the differences in the distributions of response times (RTs) of our two groups (trait-anxious and control), we performed Vincent averaging (Balota et al., 2008; Jiang et al., 2004). We first ordered each participants’ RTs (time to focus) from slowest to fastest and divided them in five equal-sized quantiles (20% of the data in each quantile). RTs of each quantile and participant were averaged individually and then averaged across participants. We explored the distributions of the data in each group using boxplots, which showed more variability and a slightly positively skewed distribution in anxious participants (Fig. 2a). We performed an ANOVA with anxiety group (control vs trait-anxious) as the between-subjects factor and quantile (quantile 1 to 5) as the within-subjects factor. This approach potentially allows the distinction between early processing (sensory) stages of visual stimuli (faces) and later evaluative stages (linking the face to the context).

### Results

#### Accuracy

The effect of emotional context on accuracy was significant (*F*(1.96, 96.13) = 50.77, *p* < 0.001, *η*^2^_p_ = 0.51; large effect size). Post hoc HSD Tukey tests showed that accuracy in all the three conditions differed significantly (*p*s < 0.05; see Table 1 for the percentage of incorrect responses in each condition).

**Table 1:** Percentage of incorrect responses for trait-anxious and control participants in each condition

	Incorrect responses (%)			
	Trait-anxious group		Control group	
	Mean	SD	Mean	SD
Context fear	25.253	10.669	29.611	10.873
Context happy	28.655	12.382	31.807	11.392
Context neutral	21.600	11.165	24.092	10.301
Distractor fear	29.473	12.459	33.045	10.440
Distractor happy	18.621	10.294	20.607	10.762
Distractor neutral	27.413	11.741	31.858	12.105

The upper section of the table refers to the percentage of incorrect responses for fearful, happy or neutral faces, while the lower part (distractor) refers to the percentage of incorrect responses for the congruent face (collapsed over facial expressions) when the distracting face was fearful, happy or neutral

For the distractor, the effect of emotion congruency on accuracy was significant (*F*(1.85, 90.49) = 100.79, *p* < 0.001,* η*^2^_p_ = 0.67; large effect size). Post hoc HSD Tukey tests revealed that accuracy for both fearful and neutral distractors was significantly different from accuracy for the happy distractor (*p*s < 0.05; see Table 1 for the percentage of incorrect responses in each condition).

#### Time to Focus

For the emotional context, the effect of anxiety group on RT was significant (*F*(1, 49) = 6.13, *p* = 0.016, *η*^2^_p_ = 0.11; medium effect size) with longer time to focus for anxious than control participants (Fig. 2a). The effect of emotional context on RT was also significant (*F*(1.96, 95.85) = 25.17, *p* < 0.001,* η*^2^_p_ = 0.34; large effect size). Post hoc HSD Tukey tests revealed that the RT in both fear and neutral contexts were significantly different from the RT in the happy context (*p*s < 0.05; see Table 2 for time to focus). Finally, the interaction between anxiety group and emotional context was significant (*F*(1.96, 95.85) = 6.34, *p* = 0.002, *η*^2^_p_ = 0.12; medium effect size) with post hoc HSD Tukey tests showing that in the anxious group, both fear and neutral contexts were significantly different from the happy context (*p*s < 0.05).

**Table 2:** Reaction times for trait-anxious and control participants in each condition

	Time to focus (ms)			
	Trait-anxious group		Control group	
	Mean	SD	Mean	SD
Context fear	392.167	105.265	337.500	60.398
Context happy	422.100	101.744	351.282	63.530
Context neutral	379.733	89.829	338.205	60.058
Distractor fear	415.033	108.291	354.327	73.233
Distractor happy	369.000	91.289	326.987	48.206
Distractor neutral	409.967	103.572	345.673	63.387

The upper section of the table refers to time to focus on fearful, happy or neutral faces, while the lower part (distractor) refers to time to focus on the congruent face (collapsed over facial expressions) when the distracting face was fearful, happy or neutral

For the distractor, the effect of distractor valence was significant (*F*(1.34, 65.83) = 22.74, *p* < 0.001, *η*^2^_p_ = 0.317; large effect size). Post hoc HSD Tukey tests revealed that both fearful and neutral distractors were significantly different from the happy distractor (*p*s < 0.05; see Table 2 for time to focus).

#### Vincent Averaging

Figure 2b shows the mean RTs for each quantile in each group. The ANOVA showed a significant effect of anxiety group (*F*(1, 49) = 7.9, *p* = 0.007, *η*^2^_p_ = 0.139; medium effect size), with longer RTs in the anxious group. The effect of quantile was significant (*F*(1.26, 61.93) = 708.66, *p* < 0.001, *η*^2^_p_ = 0.935; large effect size). The interaction anxiety group × quantile was also significant (*F*(1.26, 61.93) = 19.99, *p* < 0.001, *η*^2^_p_ = 0.289; large effect size). We then computed simple effects between groups for each quantile to test if RT differences were specific to longer times to focus, as suggested by the Vincentized distributions. The first two quantiles did not differ significantly between groups (*t*(49) = 1.18, *p* = 0.24, *d* = 0.33 for quantile 1; *t*(49) = 1.73, *p* = 0.09, *d* = 0.48 for quantile 2). The three last quantiles were significantly different between groups (*t*(49) = 2.43, *p* = 0.018, *d* = 0.68 for quantile 3 (medium effect size); *t*(49) = 3.11, *p* = 0.003, *d* = 0.87 for quantile 4 (large effect size); *t*(49) = 3.93, *p* < 0.001, *d* = 1.10 for quantile 5 (large effect size)). These results suggest that the difference in RTs between groups is pronounced for longer RTs.

#### Reaction Times for the Transitions

**Fig. 3:**
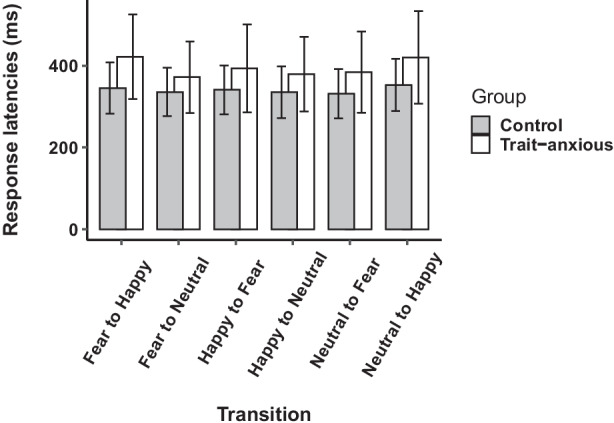
Response latencies (time to focus; RTs) for the 6 transitions in each group

For the transitions, the effect of anxiety group on RT was significant (*F*(1, 49) = 6.12, *p* = 0.016, *η*^2^_p_ = 0.11; medium effect size), as well as the effect of condition on RT (*F*(3.98, 194.97) = 13.72, *p* < 0.001, *η*^2^_p_ = 0.218; large effect size). The interaction between group and condition on RT was also significant (*F*(3.98, 194.97) = 3.77, *p* = 0.005, *η*^2^_p_ = 0.07; medium effect size). Post hoc HSD Tukey tests showed that in the trait-anxious group, RTs in a happy context following fearful or neutral contexts were significantly longer than all the other transitions. Also, RTs were significantly longer in these two conditions in the anxious group as compared to the control group (see Fig. 3).

#### Results of the Simple Response Time Task Control

For the frequentist ANOVA, none of the effects reached significance (all *p*s > 0.05). For the Bayesian ANOVA, *BFincl* = 0.075, which indicates strong evidence for H0 for the effect of emotion (Schönbrodt & Wagenmakers, 2018; Table 1), thus confirming that the faces did not differ with regard to their low-level properties, or at least that these differences were not sufficient to attract participant’s attention and bias the outcome measures.

## Discussion

The present study investigated the influence of emotional context in a modified version of the dot-probe task. We used eye-gaze as an index of attentional biases and recorded the timing of the first saccade toward emotional faces in three different emotional contexts (fearful, happy and neutral) in groups of high and low trait-anxious participants.

First, we show that effects of anxiety appear in analysis of response times (time to focus on the instructed emotion), and not accuracy. We report two main findings and will interpret our results within the framework of anxiety-related attentional biases and Bonanno and Burton’s model of regulatory flexibility (2013). First, anxious participants exhibited slower RTs than control participants, regardless of emotional context or distractor valence. Vincent averaging confirmed that this effect was restricted to longer RTs: for short RTs, there was no significant difference between groups. Second, anxious participants were slower to focus on happy faces (positive context) than neutral or fearful faces. They were however not influenced by the valence of the distractor. This was confirmed in the analysis of transitions between emotions, where RTs were slower in trait-anxious individuals for the happy context following either neutral or fearful contexts. The control study showed no effects of emotion, confirming that the low-level properties of the faces are unlikely to have impacted our outcome measures.

We compared mean RTs between groups as well as for different quantiles. In general, highly anxious participants displayed longer RTs than low-anxious participants, especially in the last quantiles (corresponding to longer RTs). We did not observe any biases to fearful faces in our study. Previous research has reported a dissociation in neural networks engaged in the processing of emotional distractors in high trait and state anxiety (Bishop et al., 2006). While high state anxious individuals were characterized by amygdala and superior temporal responses during low perceptual load processing, high trait-anxious participants showed a decrease in the prefrontal cortex associated with the processing of threat distractors. This was correlated with lower self-reported scores of attentional control and suggests diminished cognitive control in the presence of threat-related distractors. Importantly, this reduced cognitive control in trait anxiety was confirmed in tasks without emotional material (Bishop, 2008) and is accompanied by delayed RTs in identification tasks. This impoverished control may in part explain why high trait-anxious participants exhibited longer RTs in our task, which could reflect reduced executive control enhanced by the stressor (Pacheco-Unguetti et al., 2010).

The fact that RT differences between the two groups were prominent for later quantiles suggests that the timing of this processing is crucial to understand attentional biases in anxiety. Differences in later quantiles suggest an effect at an evaluative stage, the ability to decide which emotional face fits the context, and not at an earlier stage, such as a sensory stage affecting the ability to detect an emotional face. If that was the case, the earliest responses should be different as well. It is possible that in the earliest quantiles, the context is very salient and is processed without deficit by anxious individuals, whereas in later quantiles, the context is less salient and attentional biases are manifest.

In our study, attentional biases were restricted to the positive faces, as time to focus was longer for the positive context than neutral or negative contexts in the trait-anxious group. Since this effect was not present when the distractor was positive, we hypothesize that this is not an attentional bias away from positive stimuli but is likely a difficulty in attending toward positive information. This was confirmed in the analysis of transitions between emotional contexts, where RTs were longer for the happy context, whether it followed the neutral or the fearful context. These results are consistent with earlier reports in which anxiety vulnerability has been associated with reduced attention allocation to positive cues (Taylor et al., 2011). Taylor et al (2011) reported that attentional training to enhance attention towards positive stimuli resulted in less subsequent stress-related responses. Similarly, participants instructed to direct their attention toward positive stimuli demonstrated less frustration during the completion of a stressful anagram task (Johnson, 2009), again showing the adaptive role of positive emotions in response to stressors. Twivy et al. (2021) have also found in an affective flexibility task that high-anxious participants were slower to shift attention towards the affective aspects of positive material. Crucially, this impaired processing of positive information was associated with increasing levels of self-reported anxiety over a 7-week period. Taken together, these findings suggest that disruptions to the processing of positive information may be an important cognitive aspect of anxiety vulnerability.

From the perspective of decoding contextual cues, the importance of positive emotions in coping with chronic stress has also been highlighted (Folkman and Moskowitz, 2000), and difficulties in experiencing positive emotions are frequently reported in depression (Gruber et al., 2010). As discussed earlier, we know that psychopathological states such as depression or anxiety are often associated with context insensitivity (see Bonanno & Burton, 2013; Larson et al., 2007). Three of our findings provide evidence for context insensitivity in anxious individuals. First, we replicate an inverse association between the CSI-cue absence and measures of trait anxiety. Second, high-anxious individuals displayed slower times to focus on a target facial expression than low-anxious participants. This finding could reflect a difficulty in linking emotional expressions (at the perceptual stage) and responses to contextual demands (actually orienting one’s gaze to the instructed emotion). This interpretation is supported by the difference between anxious and control participants being prominent for longer RTs (later quantiles after Vincent averaging), hence at the evaluation stage and not during the perception stage. Finally, the effect of context insensitivity was restricted to positive emotions, therefore suggesting that anxious participants have difficulty engaging towards positive emotions in a positive context. If the context is not correctly decoded or is delayed (as reflected by longer response times in the happy context), it is likely to impact the other components of emotion regulation.

The present study had some limitations that should be taken into consideration and guide future research on emotional processing in anxiety using measures of context sensitivity. First, it is not clear to which extent our reward structure is a necessary feature of the paradigm or to which extent it was effective in generating mild anxiety. In addition to that, cash rewards may be less of an incentive in wealthier populations, which could have an impact on generalizability. If there is an effect of reward, it could be driven by the stress of losing money and this effect could be modulated by individual needs. Future investigations could consider the effect of reward and how it is modulated by individual factors. Second, we did not consider participants with clinical levels of anxiety, which could be an interesting avenue for future research. As such, we lacked information about participants’ mental health history and whether they had a diagnosis of anxiety. Third, since there is an interval of a maximum of 1 week between the assessment of trait anxiety and the experimental phase, there is a possibility that anxiety levels could have been significantly different between those two points in time. Although trait anxiety has shown stability over time in a 2-year (Usala & Hertzog, 1991) or 12-month (Werner et al., 2022) interval, we cannot exclude that external life events could have differentially impacted participants’ anxiety levels during the experimental phase.

While anxiety-related attentional biases have been widely reported, these have often been based on dot-probe paradigms based on analysis of reaction times where serious questions have been raised about reliability (Parsons et al., 2016). Our use of gaze to assess biased attention is likely to provide a more ecologically valid measure and reliable assessment. Our results support the growing evidence that processing of affectively positive information may be an important part of the development of anxiety vulnerability. We have also attempted in this work to investigate biased attention within a broader framework of sensitivity to context. Further work is required to find better ways of measuring context sensitivity in paradigms that can assess biases in attention and other cognitive processes.

## Data Availability

MATLAB scripts and data are freely available on the Open Science Framework at https://osf.io/qjyt9/
